# Supplementary material for: Proteomic Mapping of the Interactome of KRAS Mutants Identifies New Features of RAS Signalling Networks and the Mechanism of Action of Sotorasib
Source: Cancers (Basel). 2023 Aug 17;15(16):4141. doi: 10.3390/cancers15164141 (PMC10452836; doi:10.3390/cancers15164141)

Figure S1

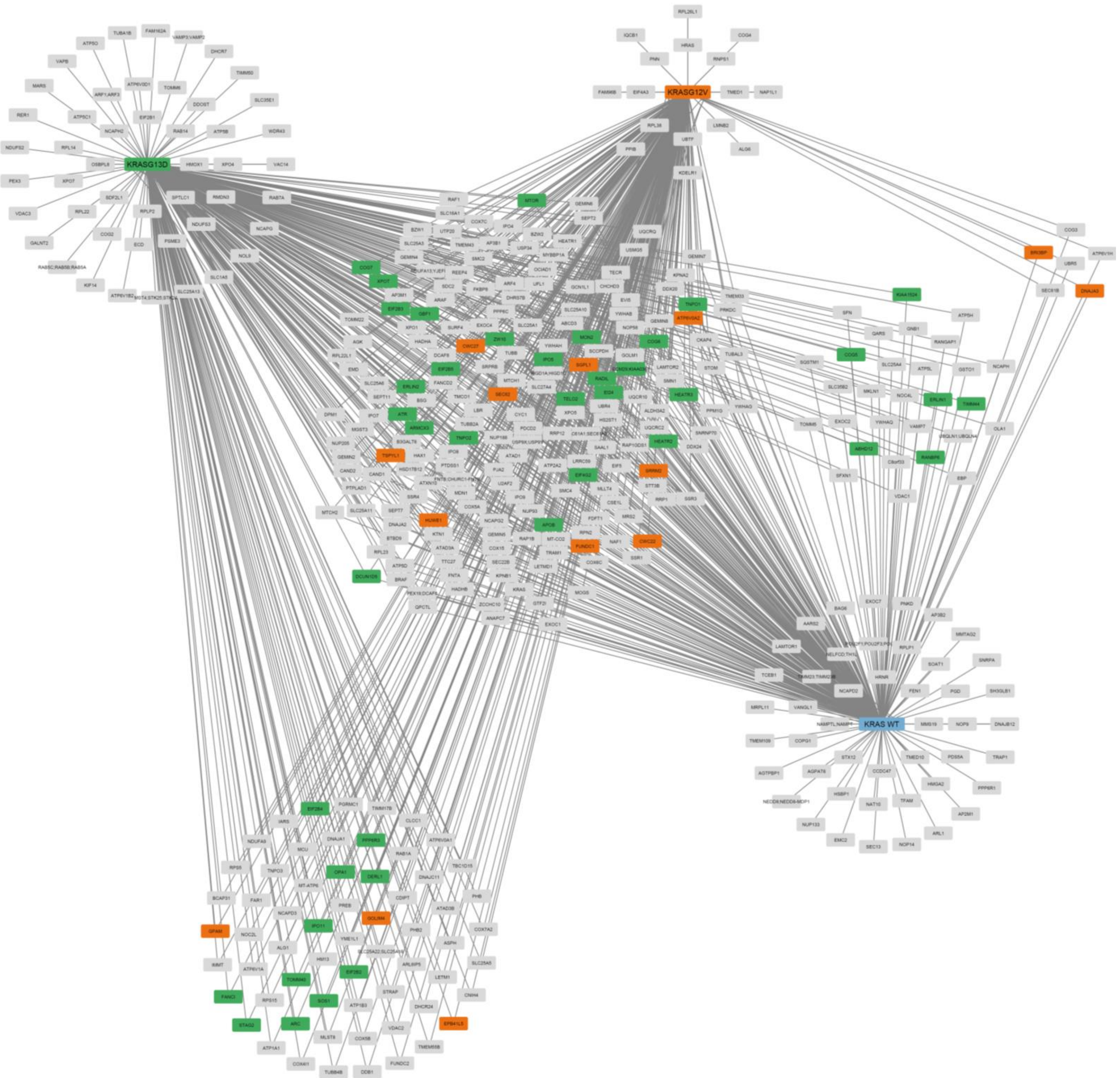

Supplementary Figure S1: Specific protein interaction network of KRAS isoforms in Hek293 cells, demonstrating proteins exclusive to one isoform (KRAS WT in blue, KRAS G13D in green, and KRAS G12V in orange). Shared proteins coloured green have greater affinity for the KRASG13D mutant when compared to other conditions or specific interactors of all KRAS isoforms, proteins coloured orange have greater interaction/binding affinity for the KRASG12V mutant when compared pairwise to the other 3 conditions or specific interactors for all 3 KRAS isoforms. Protein interaction networks of KRAS were reconstructed using Cytoscape.

Figure S2

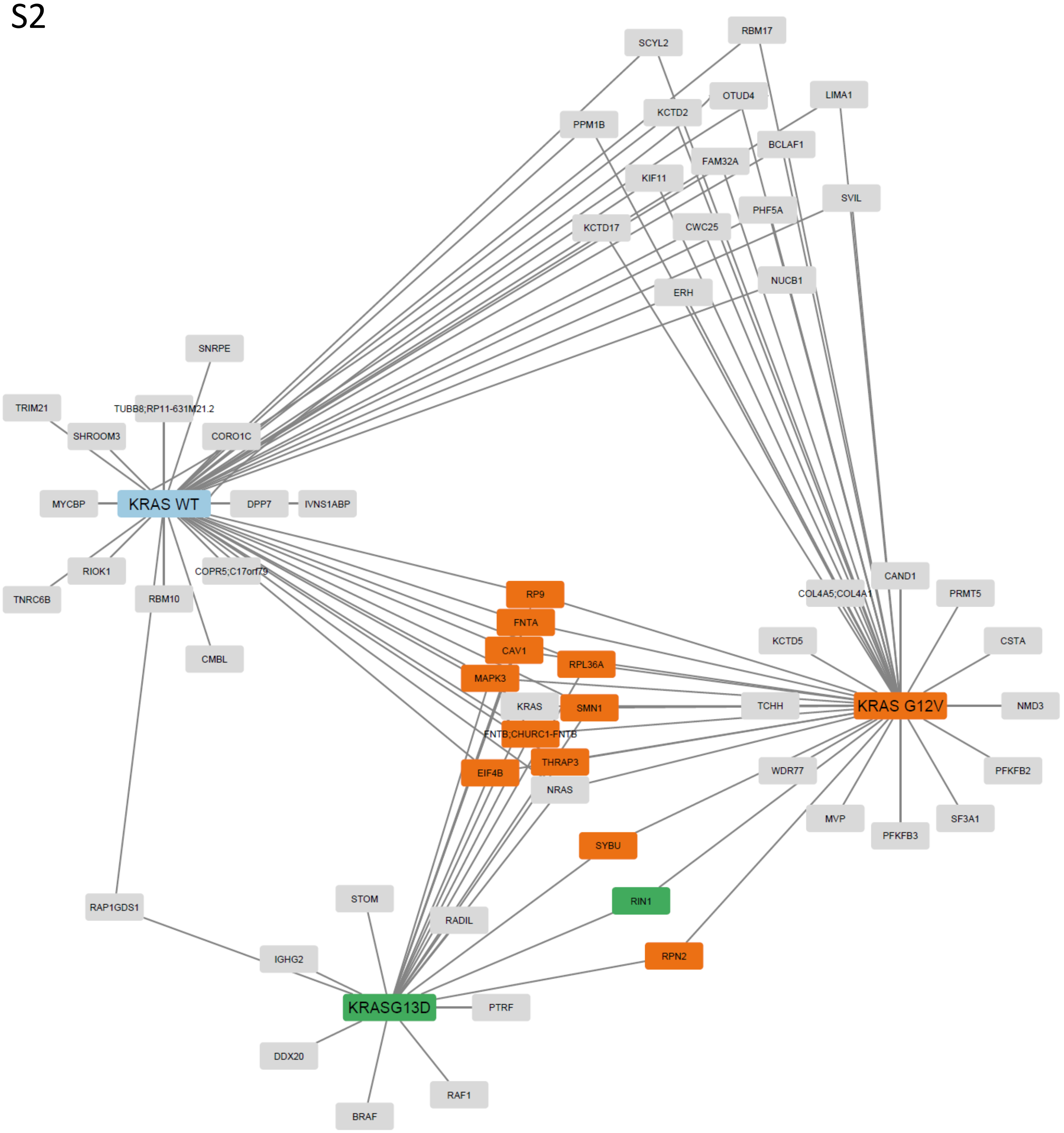

Supplementary Figure S2: Specific protein interaction network of KRAS isoforms in HKe-3 cells, demonstrating proteins exclusive to one isoform (KRAS WT in blue, KRAS G13D in green, and KRAS G12V in orange). Shared proteins coloured green have greater affinity for the KRASG13D mutant when compared to other conditions; proteins coloured orange have greater interaction/binding affinity for the KRASG12V mutant when compared pairwise to the other 3 conditions or specific interactors for all 3 KRAS isoforms. Protein interaction networks of KRAS were reconstructed using Cytoscape.

Figure S3

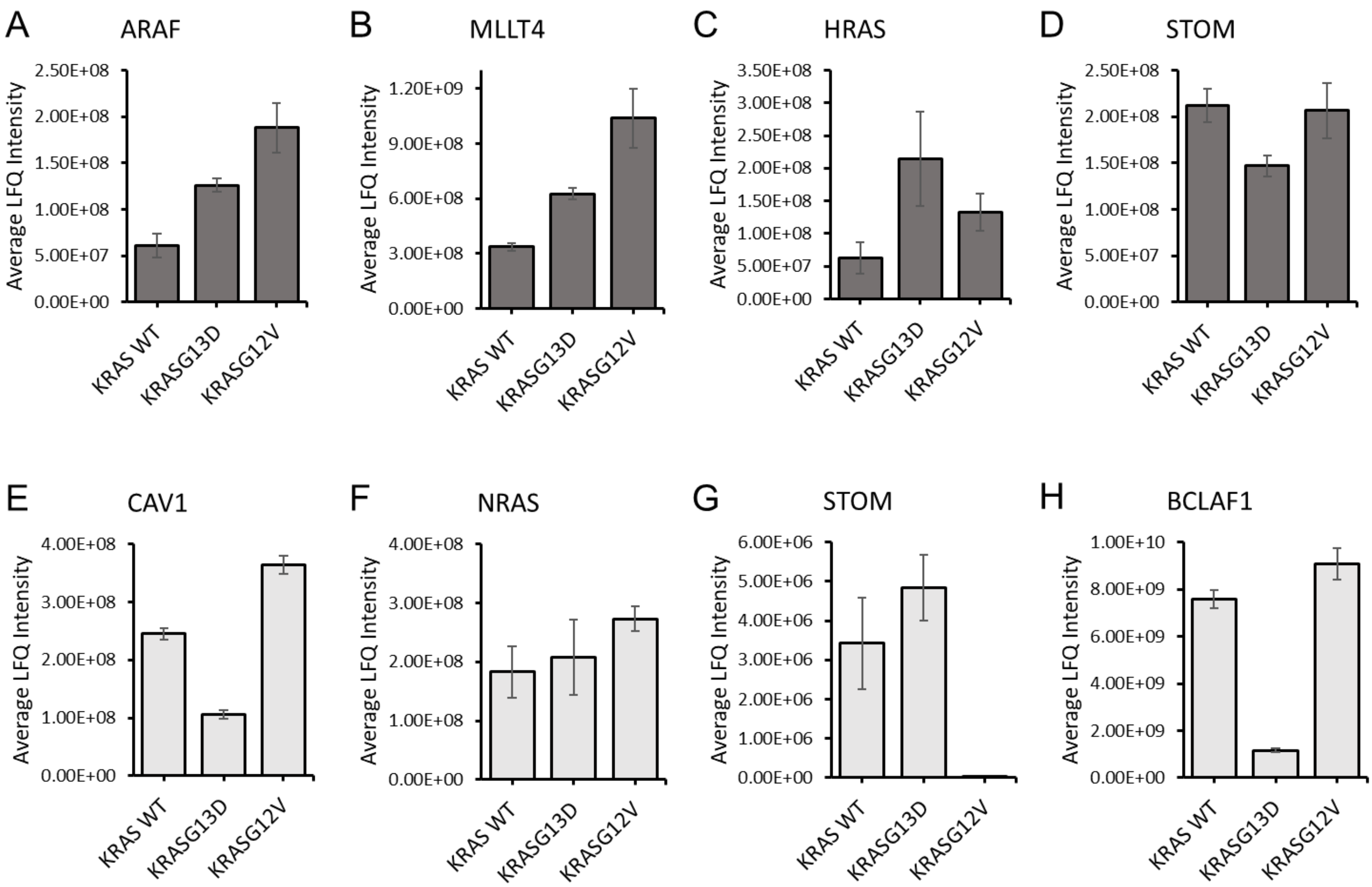

Supplementary Figure S3. AP-MS identify KRAS proteins differential interacting proteins A-D) graphs show the average LFQ values in HEK293 cells of the indicated proteins in immunoprecipitates of KRAS WT, KRASG13D or KRASG12V as indicated (n=3). Error bars show SD. E-H) graphs show the average LFQ in HKe-3 cells of the indicated proteins in immunoprecipitates of KRAS WT, KRASG13D or KRASG12V as indicated (n=3). Error bars show SD.

Figure S4

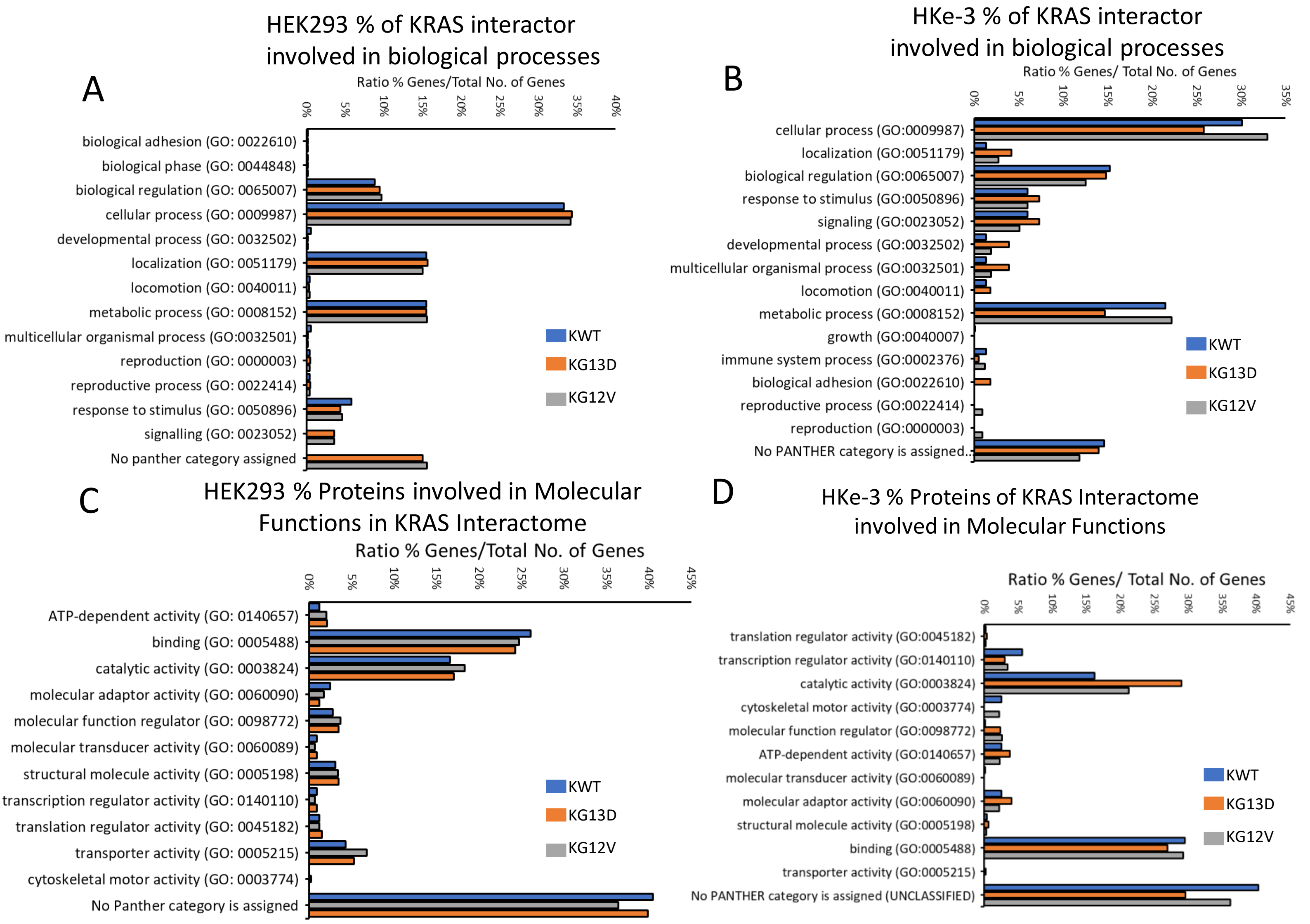

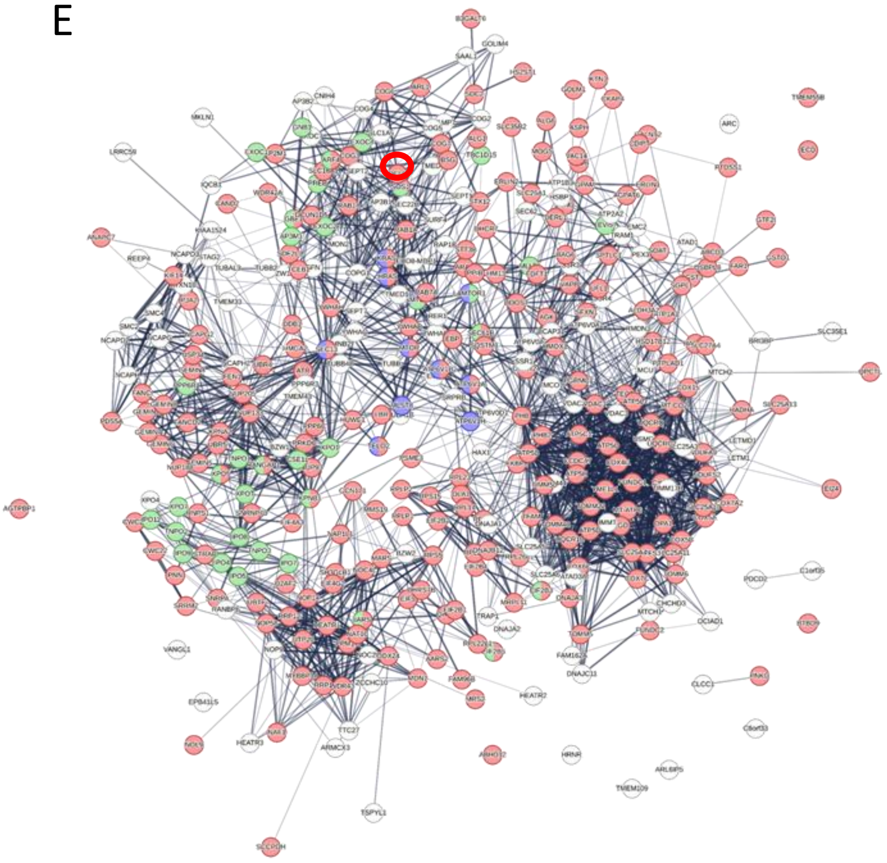

Supplementary Figure S4. Bioinformatic functional analysis of AP-MS data in HKE393 and HKe-3cells. A) Representation of GO terms enriched in the specific proteins bound to KRAS WT, KRASG13D or KRASG12V in HEK293 cells obtained using Panther analysis for biological processes. B) Representation of GO terms enriched in the specific proteins bound to KRAS WT, KRASG13D or KRASG12V in HEk-3 cells obtained using panther analysis. C) Representation of GO terms enriched in the specific proteins bound to KRAS WT, KRASG13D or KRASG12V in HEK293 cells obtained using panther analysis for molecular functions. D) Representation of GO terms enriched in the specific proteins bound to KRAS WT, KRASG13D or KRASG12V in HEk-3 cells obtained using panther analysis. E) Pathway reconstruction and cluster analysis performed with StringDB show HEK293 specific protein interactors of KRAS that are exclusive to the HEK293 cell line. Red halo indicates KRAS node. Edges represent confidence, line thickness indicates the strength of data support. K-means clustering was performed for 3 clusters. Proteins coloured red are involved in metabolic processes. Proteins coloured blue are involved in mTOR signalling pathway. Green proteins are involved in GTPase binding.

Figure S5

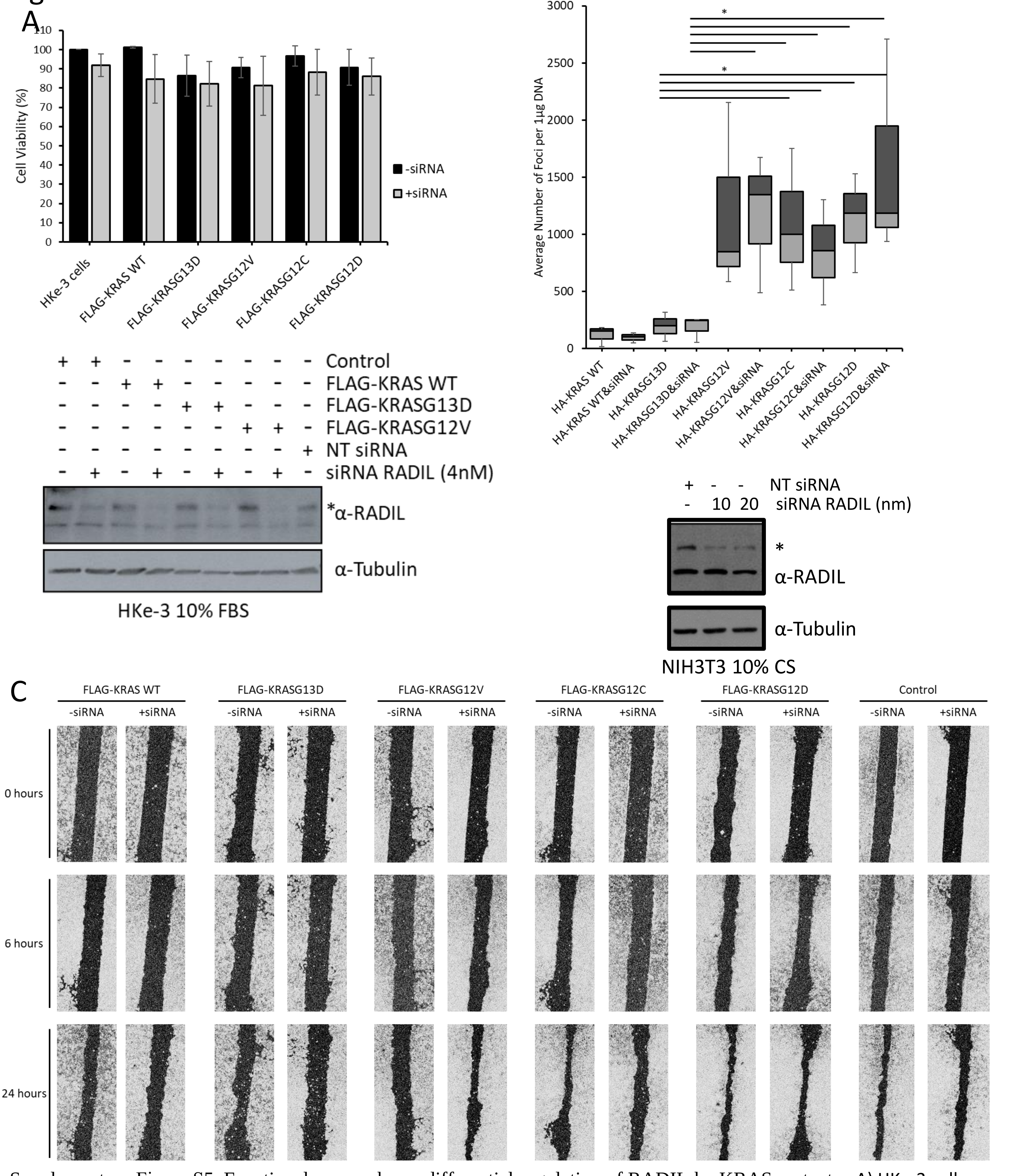

Supplementary Figure S5. Functional assays shows differential regulation of RADIL by KRAS mutants A) HKe-3 cells were transfected with empty vector or the indicated FLAG-tagged KRAS constructs and/or siRNA against RADIL (4nM). Cell viability was a measured used MTS assay 24hours after transfection (n=3). Lower panel, HKe-3 cells were transfected with RADIL 4nM siRNA-RADIL and the indicated KRAS constructs or empty vector in parallel with cells used in figure 5F. Cells were lysed 48 hour after transfection and extract were blotted with the indicated antibodies. B) NIH3T3 were transfected with 200ng of FLAG-KRAS WT (WT), -KRASG13D (G13D), -KRASG12V (G12V), -KRASG12C (G12C), -KRASG12D (G12D), or empty vector and/or RADIL siRNA (20nM). 14 days after transfection plates were fixed and stained with Giemsa and macroscopic foci were counted. Numbers show average number of foci per 1ug DNA +/- SD (n=3). Lower panel, NIH3T3 cells were transfected with 20nM siRNA-RADIL and the indicated constructs in parallel with the cells used for foci assay. Cells were grown for 5 days and lysates were incubated with the indicated antibodies. C) cells transfected as in A were let grow to confluency in a plate with a rubber stopper. Stoppers were removed and images were taken at the indicated times and gap closure was measured.. Percentage gap closure was quantified using ImageJ and results were normalised to 0h time point [36] The whole western blot figure can be found in Suppl. materials Original Blots and quantification for figure S5

Figure S6

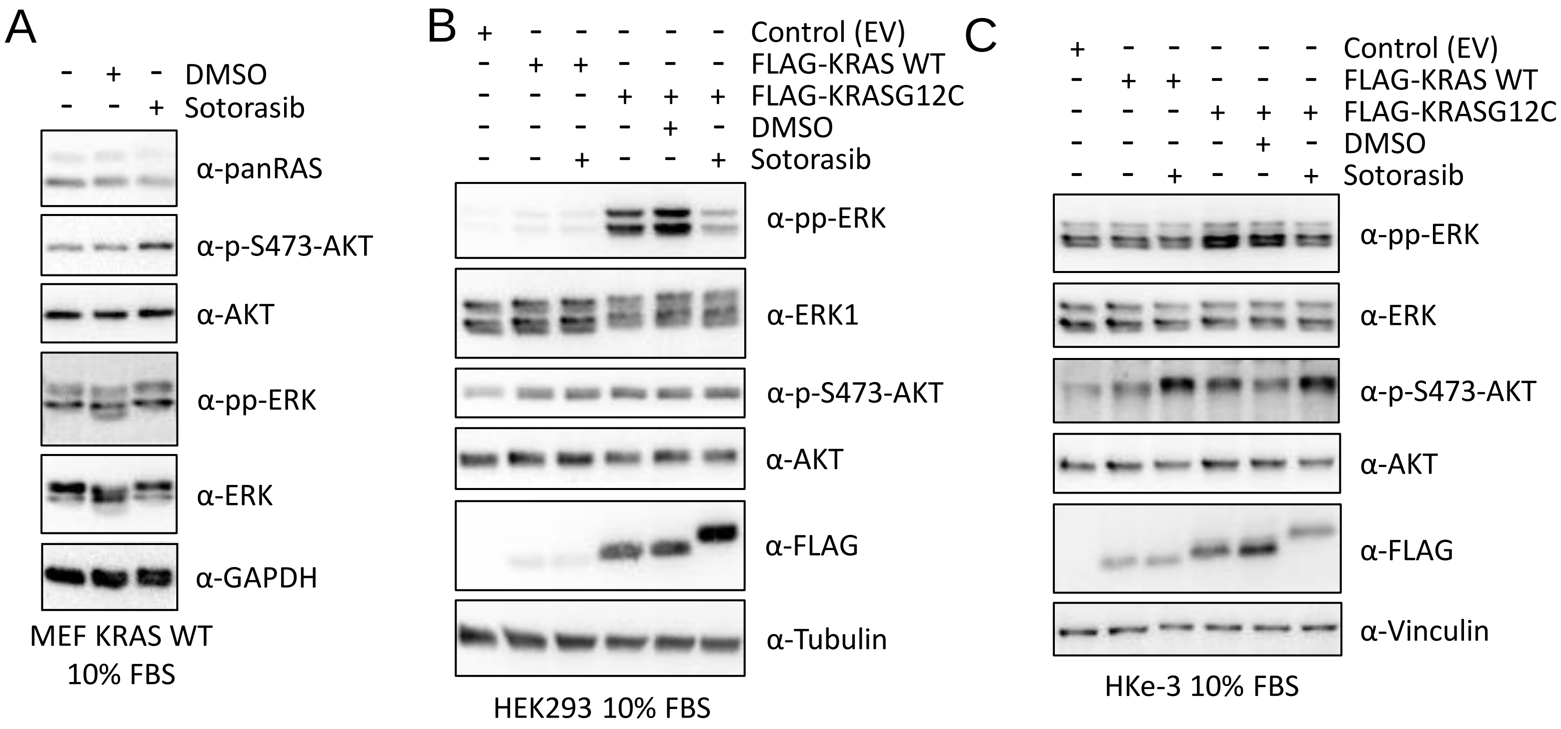

Supplementary Figure S6: Sotorasib regulates of KRAS effectors in different cells types A) MEF KRAS WT were treated with of sotorasib (5µM) for 24hours. Cells were lysed and the indicated proteins were blotted using the indicated antibodies. B) HEK293 cells were transfected with the indicated constructs. 24 hours after transfection the cells were treated with sotorasib (5µM) for 24 hour. Cell lysates were blotted with the indicated antibodies. C) HKe-3 cells were transfected as in B cell extract were detected using the indicated antibodies. . The whole western blot figure can be found in Suppl. materials Original Blots and quantification for figure S6

Figure S7

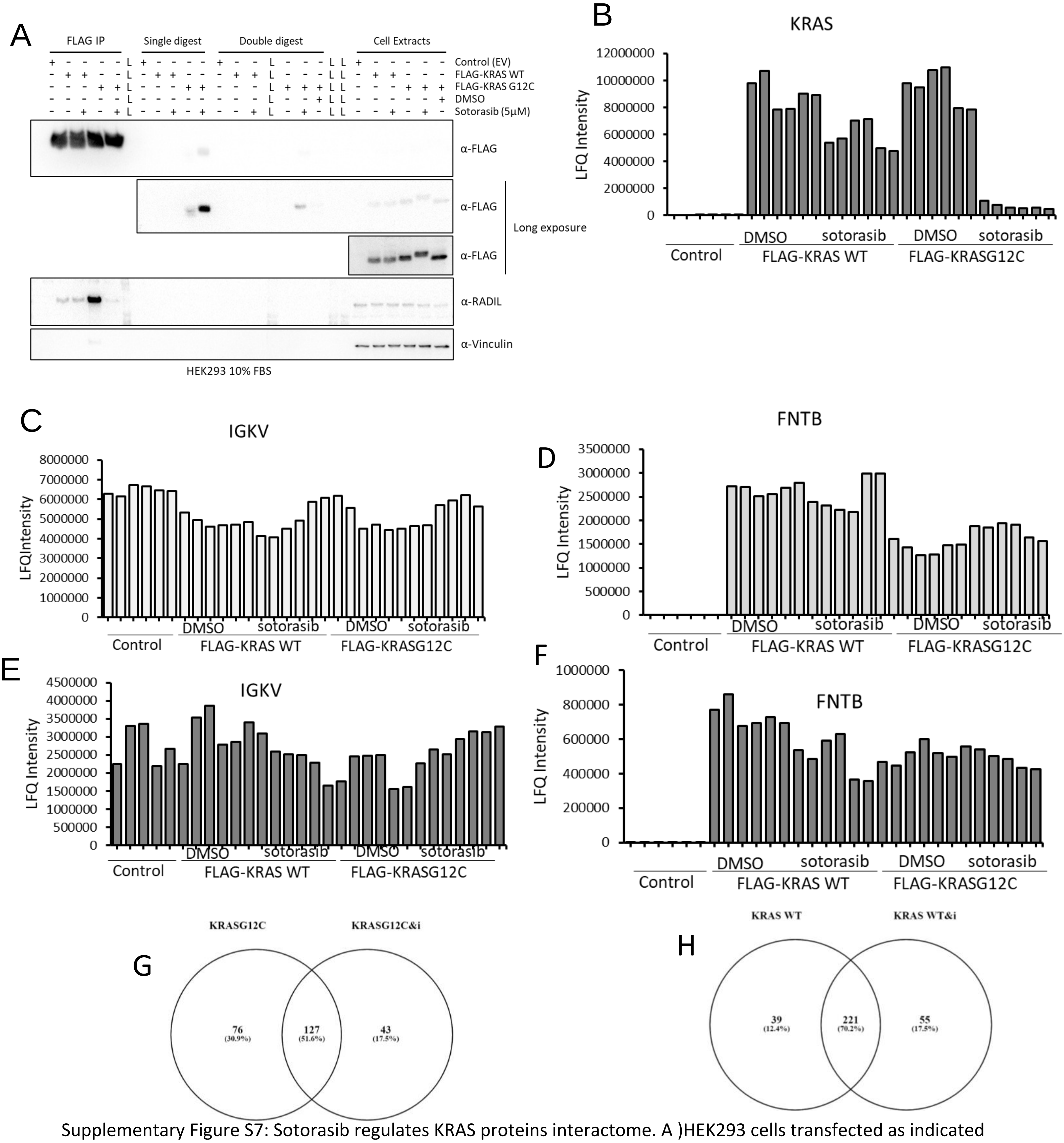

Supplementary Figure S7: Sotorasib regulates KRAS proteins interactome. A)HEK293 cells transfected as indicated were immunoprecipitated and the IP was divided in three aliquots, undigested (FLAG IP), digested with trypsin (single digest) or trypsin and LysC (double digest). The samples were western blotted with the indicated proteins. (L indicate empty lanes). . The whole western blot figure can be found in Suppl. materials Original Blots and quantification for figure S7 B-E) HEK293 cells were transfected with the indicated constructs. After 24hour cells were treated with sotorasib (i, 5μM) or DMSO for 24 hours. Cell lysates were digested with trypsin (C and D), or trypsin and LysC (B, E and F). Graph shows the LFQ intensity of all the samples of the indicated proteins. G) shows Venn diagram representation of the proteins that are identified by AP-MS to be specifically interacting with KRASG12C in the absence or presence (i) of sotorasib. H) shows Venn diagram representation of the proteins that are identified by AP-MS to be specifically interacting with KRAS WT in the absence or presence (i) of sotorasib.

figure S7 cont

I

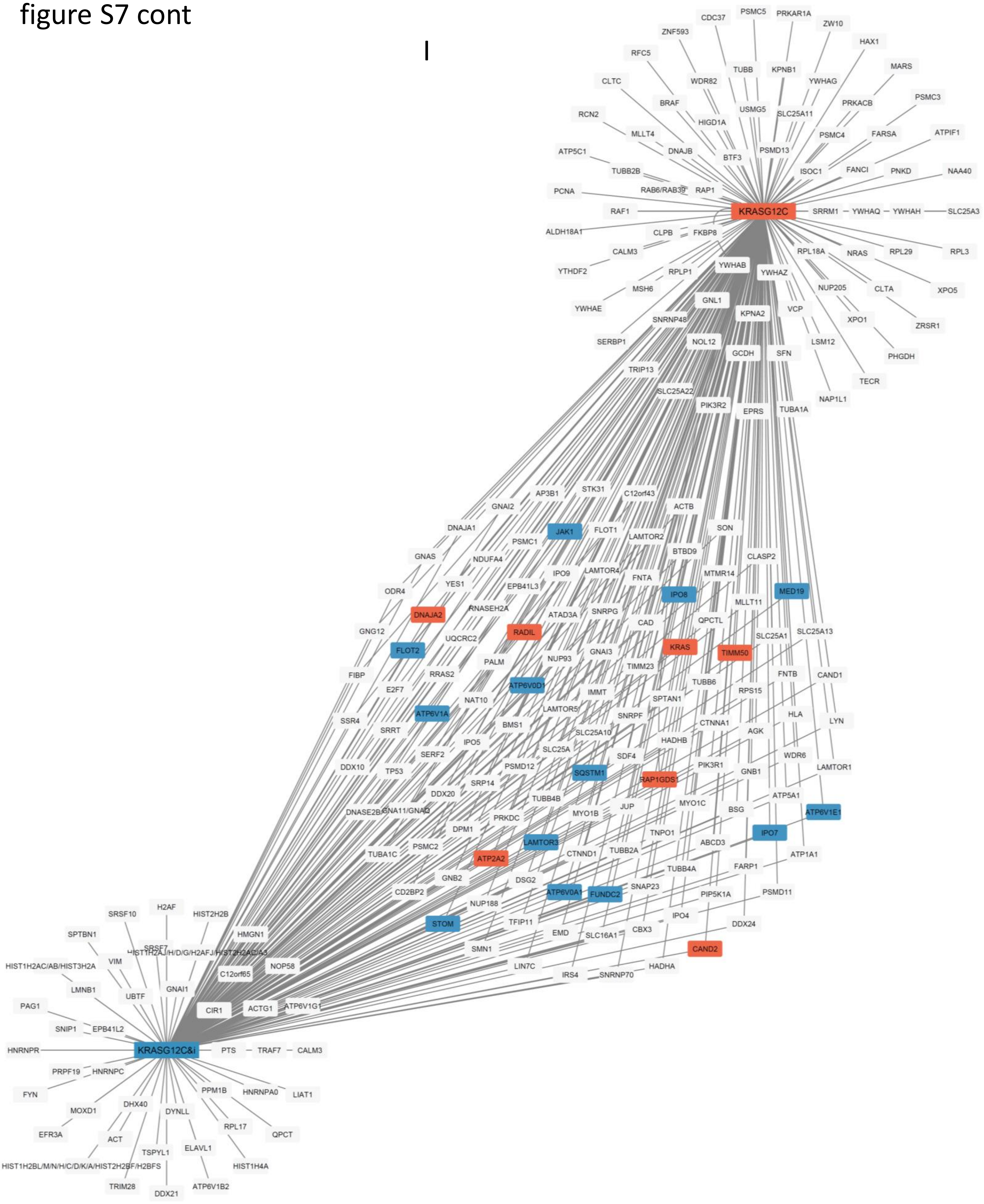

Supplementary Figure S7I: Specific protein interaction network of KRASG12C in cells treated with 5µM of sotorasib (i, blue colour) ore untreated (orange). Shared proteins coloured blue have greater affinity for the KRASG12C treated with sotorasib.; proteins coloured orange have greater interaction/binding affinity for the KRASG12C mutant in untreated cells. Protein interaction networks of KRAS were reconstructed using Cytoscape.

Figure S7 cont.

J

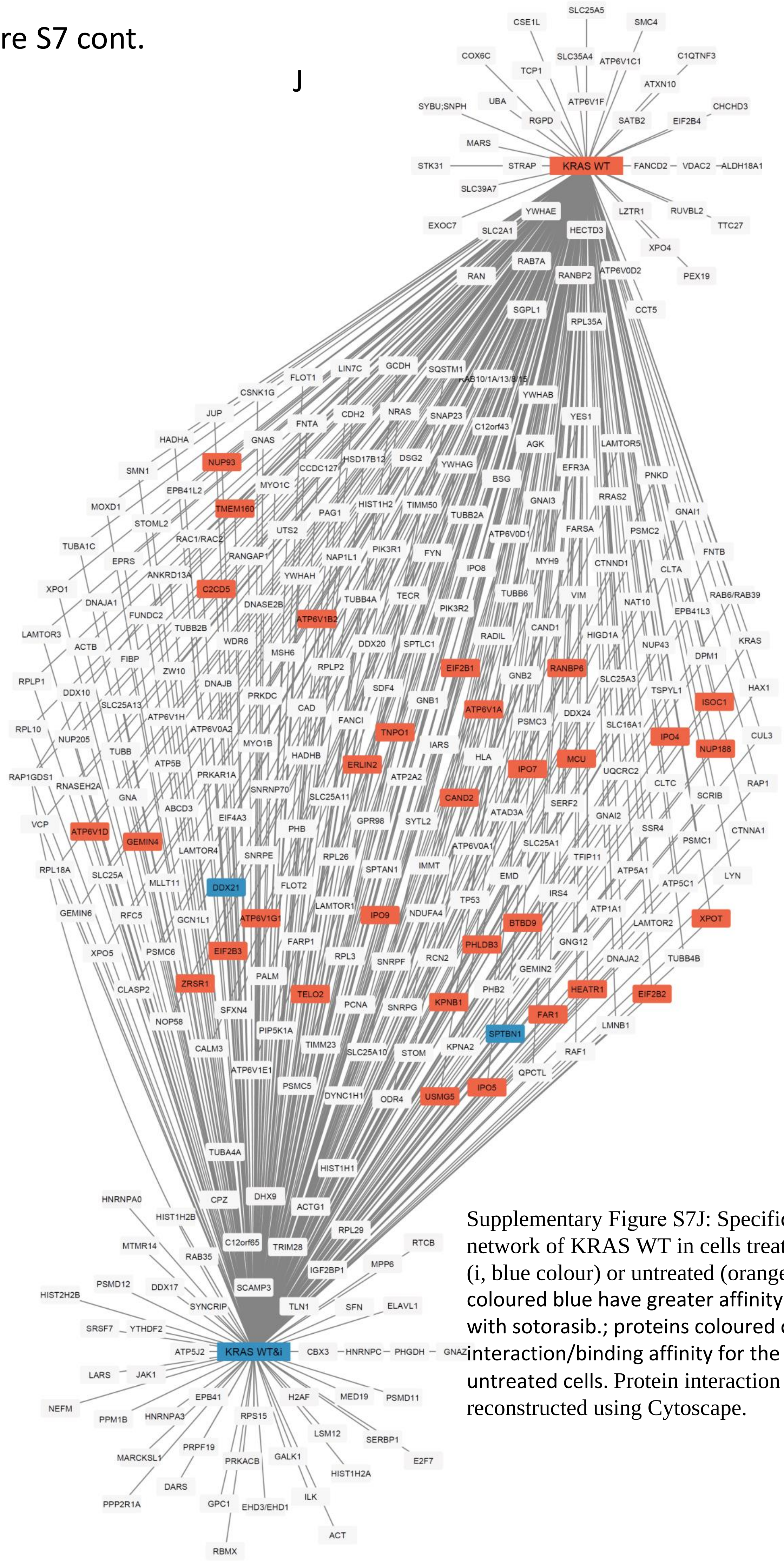

Supplementary Figure S7J: Specific protein interaction network of KRAS WT in cells treated with 5µM of sotorasib (i, blue colour) or untreated (orange). Shared proteins coloured blue have greater affinity for the KRAS WT treated with sotorasib.; proteins coloured orange have greater interaction/binding affinity for the KRAS WT mutant in untreated cells. Protein interaction networks of KRAS were reconstructed using Cytoscape.

Figure S8

A

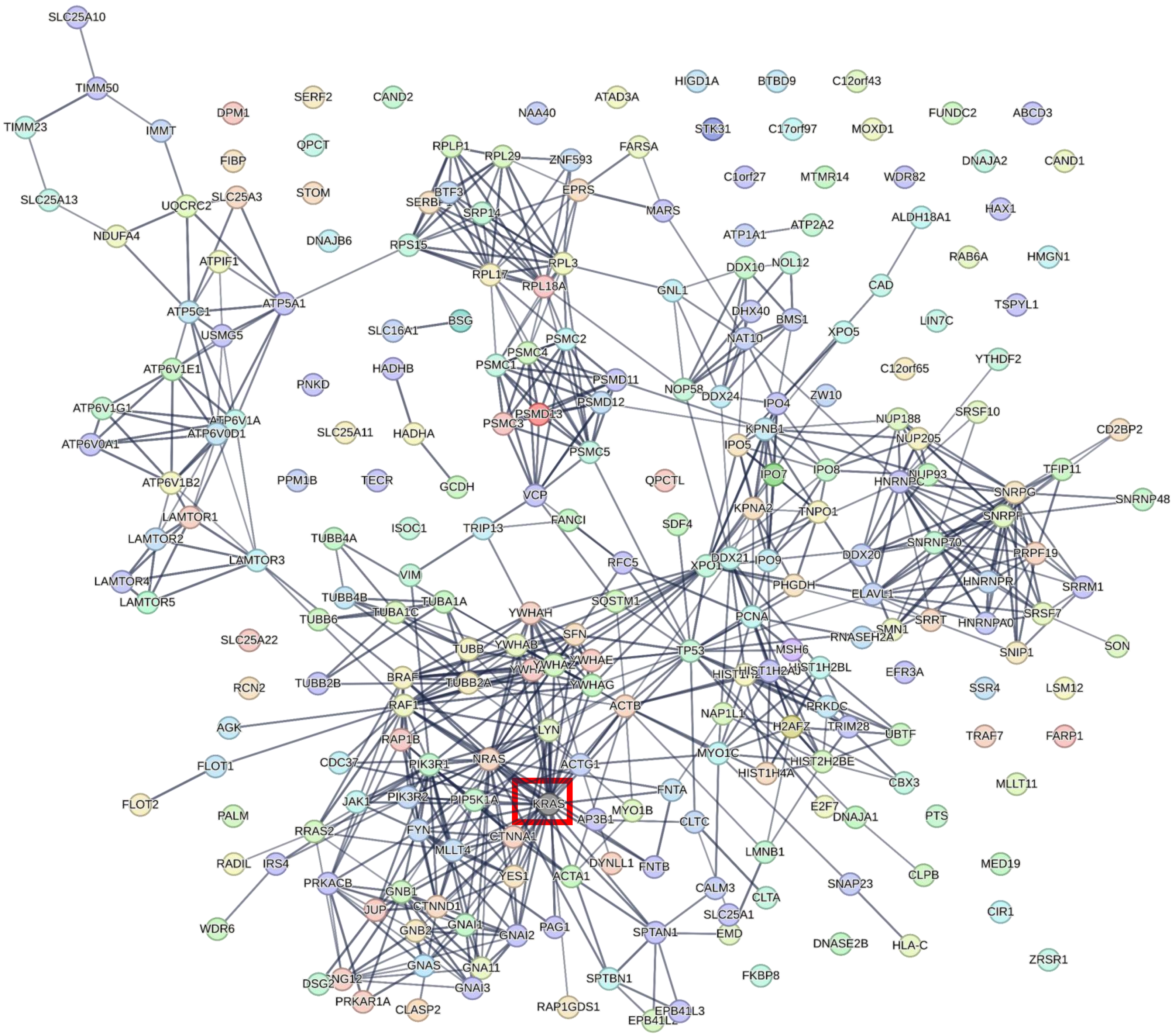

Supplementary Figure S8A: Network reconstruction generated in StringDB showing all KRASG12C specific interactome identify in FLAG-KRASG12C IPs in HEK293 cells treated with sotorasib and untreated.. Red square indicates KRAS. Edges show confidence (high confidence).

Figure S8 cont.

B

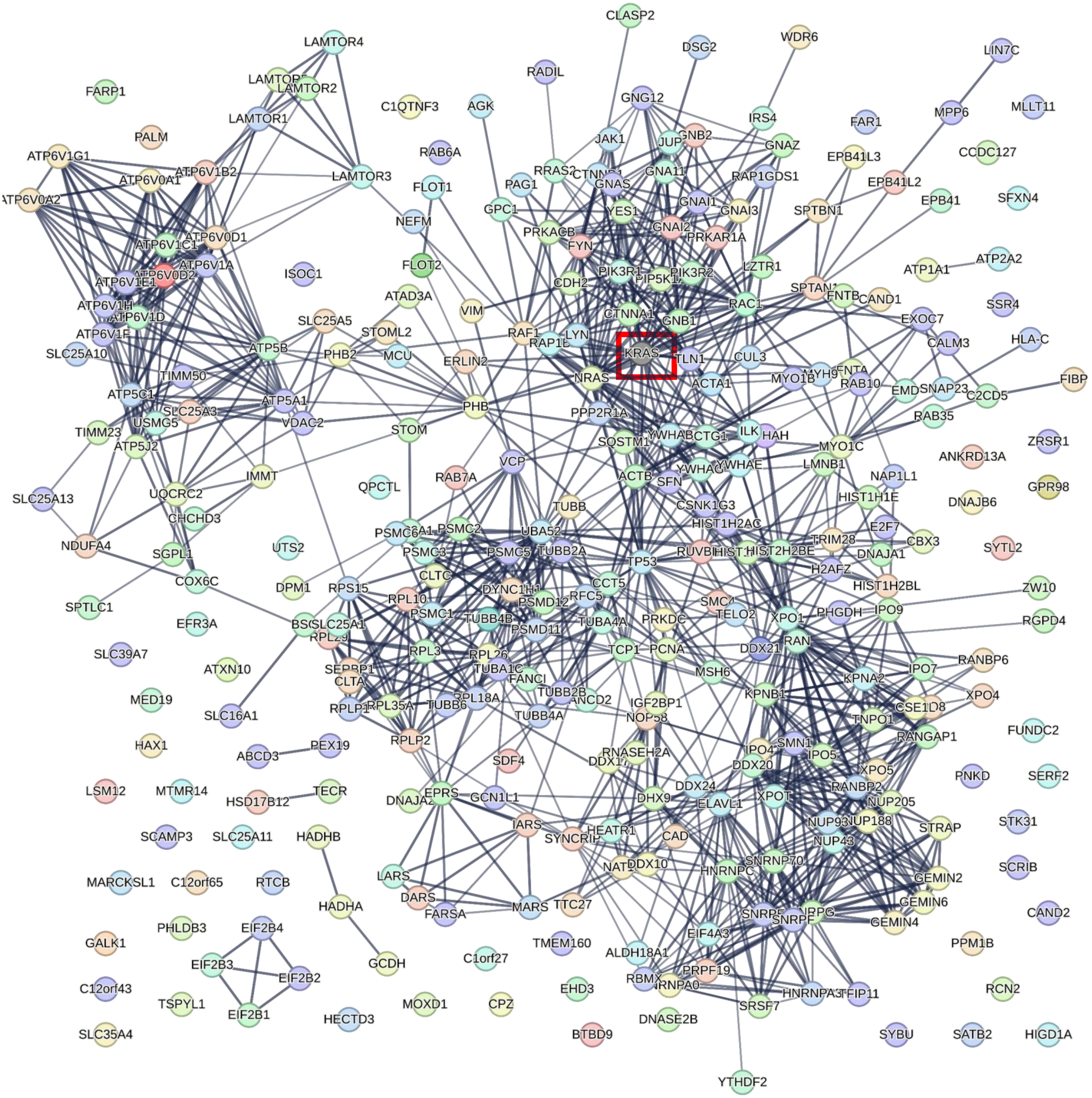

Supplementary Figure S8B: Network reconstruction generated in StringDB showing all KRAS WT specific interactome identify in FLAG-KRAS WT IPs in HEK293 cells treated with sotorasib and untreated. Red square indicates KRAS. Edges show confidence (high confidence).

Original blots and quantification

Original blots for Figure 1A

NUMBER IN ALL FIGURES ARE FOLD CHANGES OF DENSITOMERY RATIO WITH RESPECT To FIRST LINE OF THE BLOT

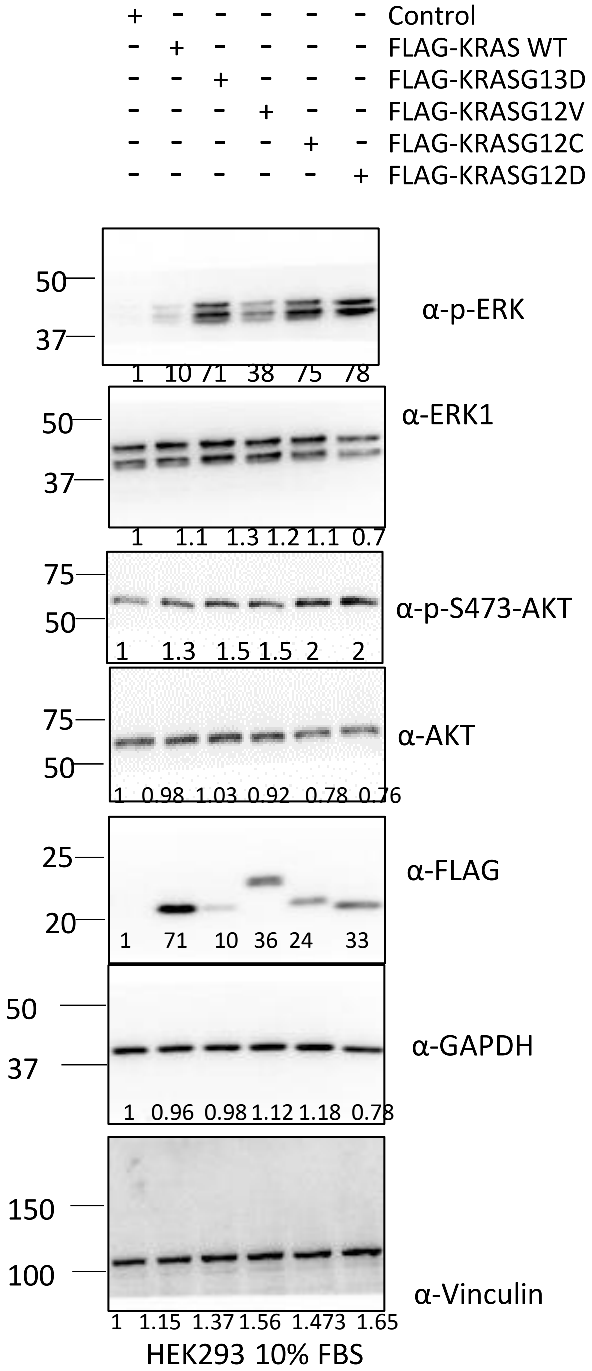

Original Blots and quantification for Figure 4

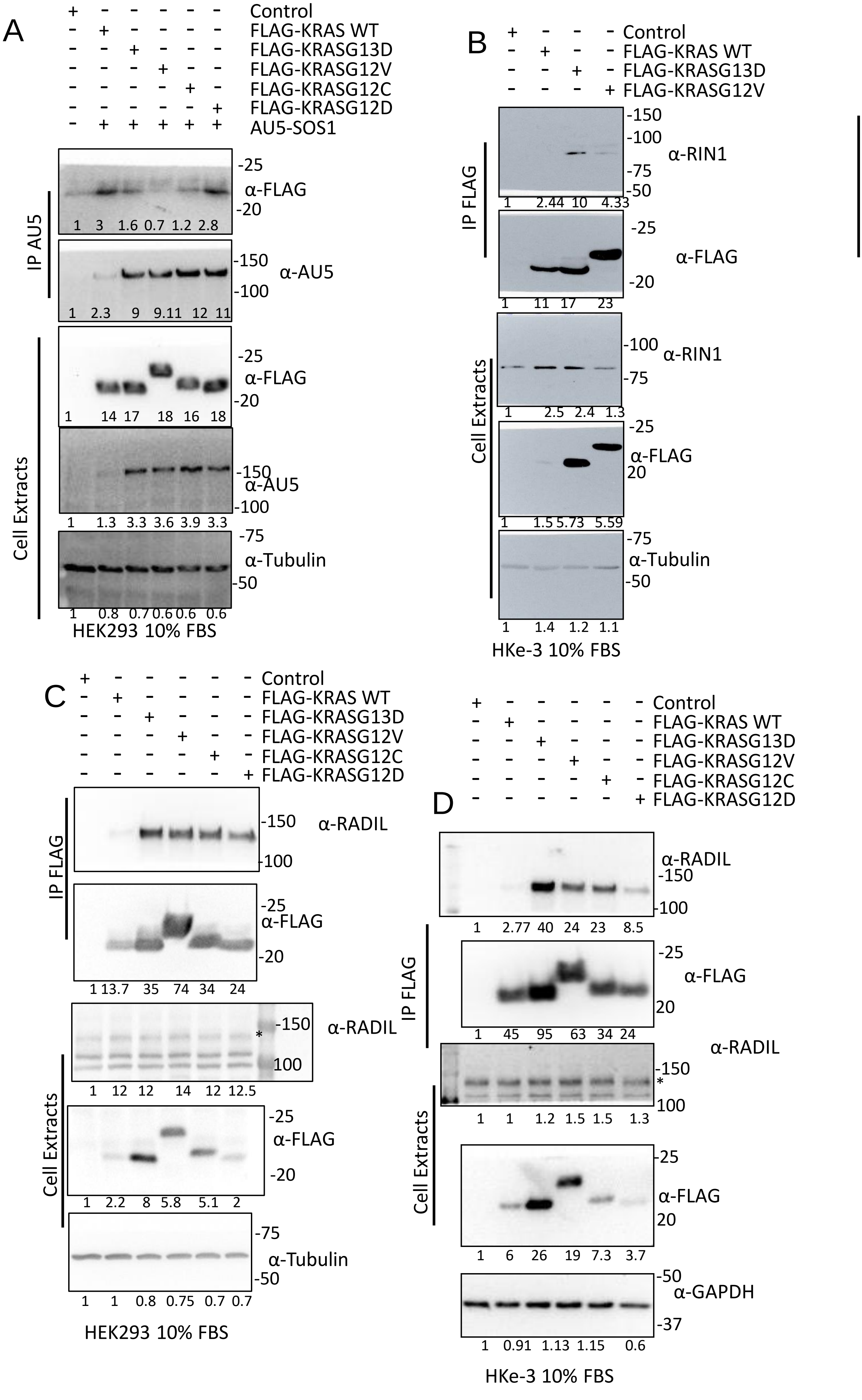

Original Blots and quantification for Figure 4

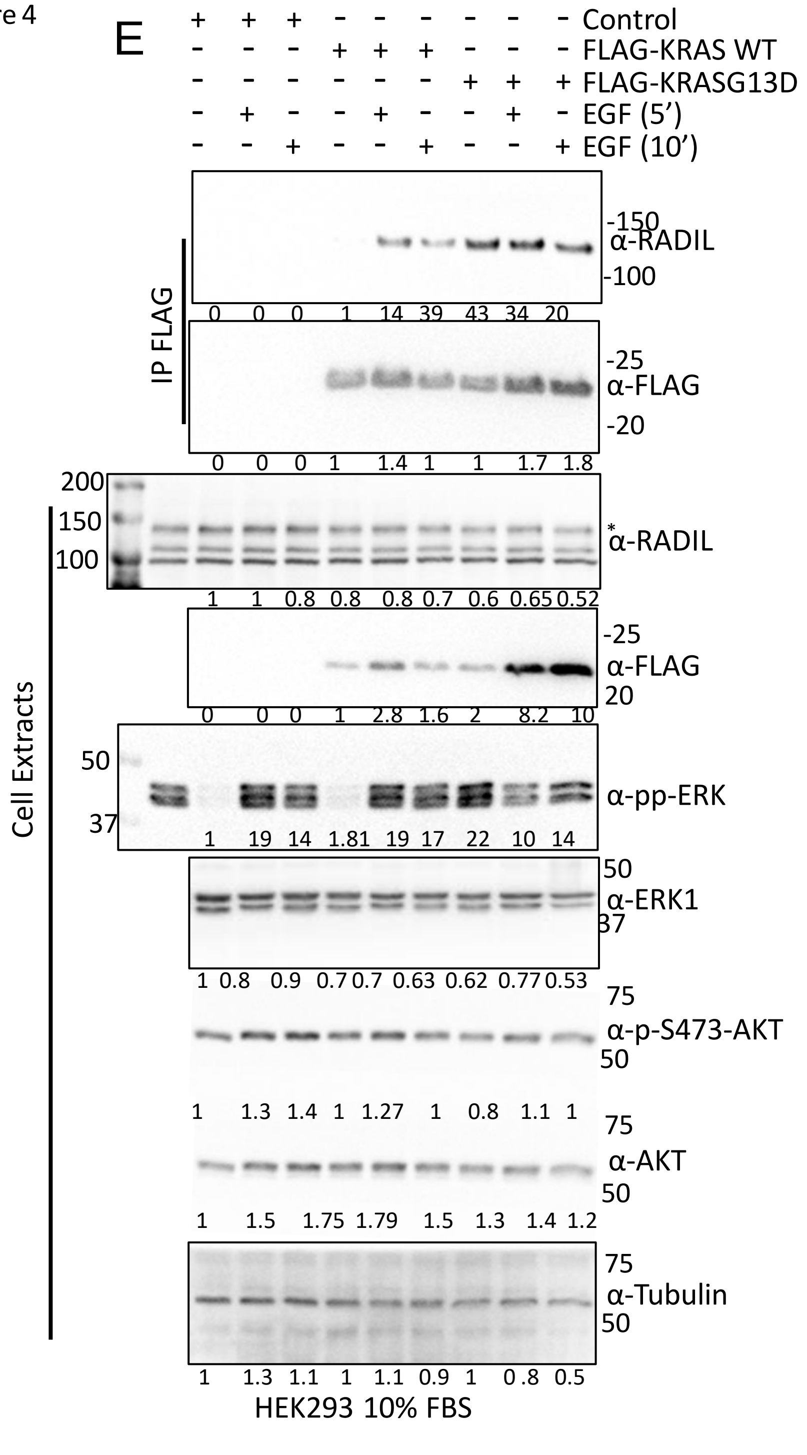

Original Blots and quantification for Figure 5

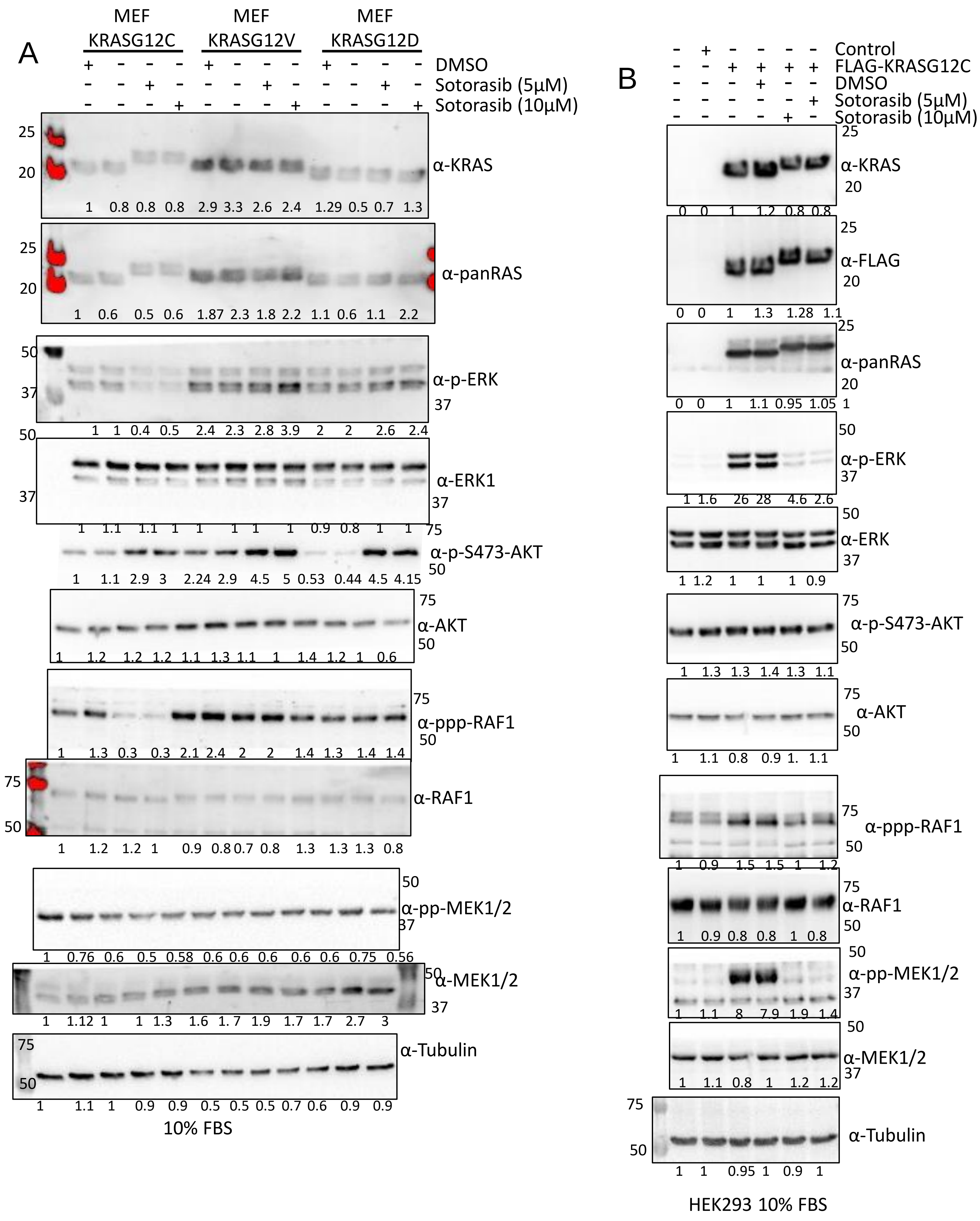

Original Blots and quantification for Figure 6 B

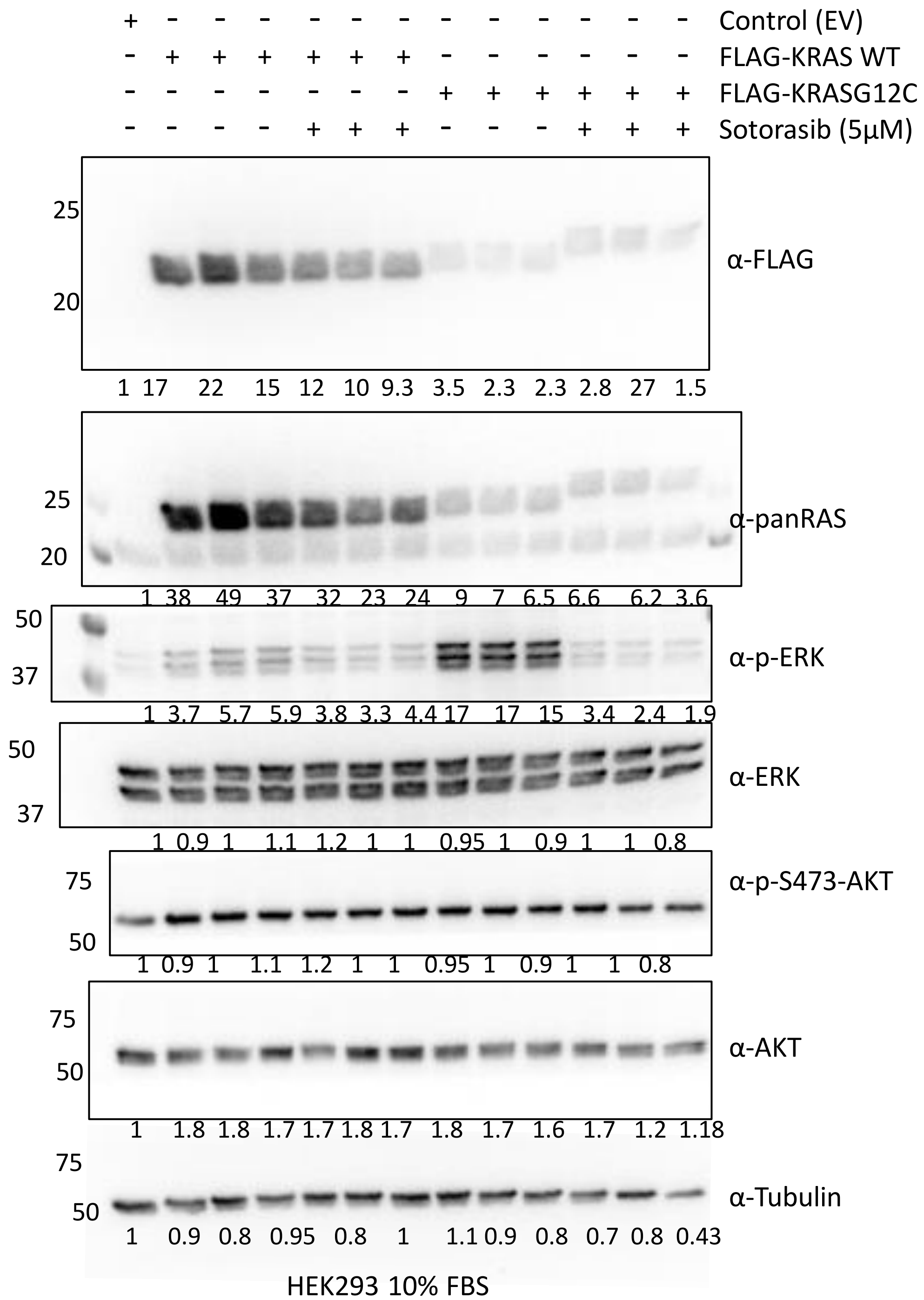

HEK293 10% FBS

Original Blots and quantification for Figure 8

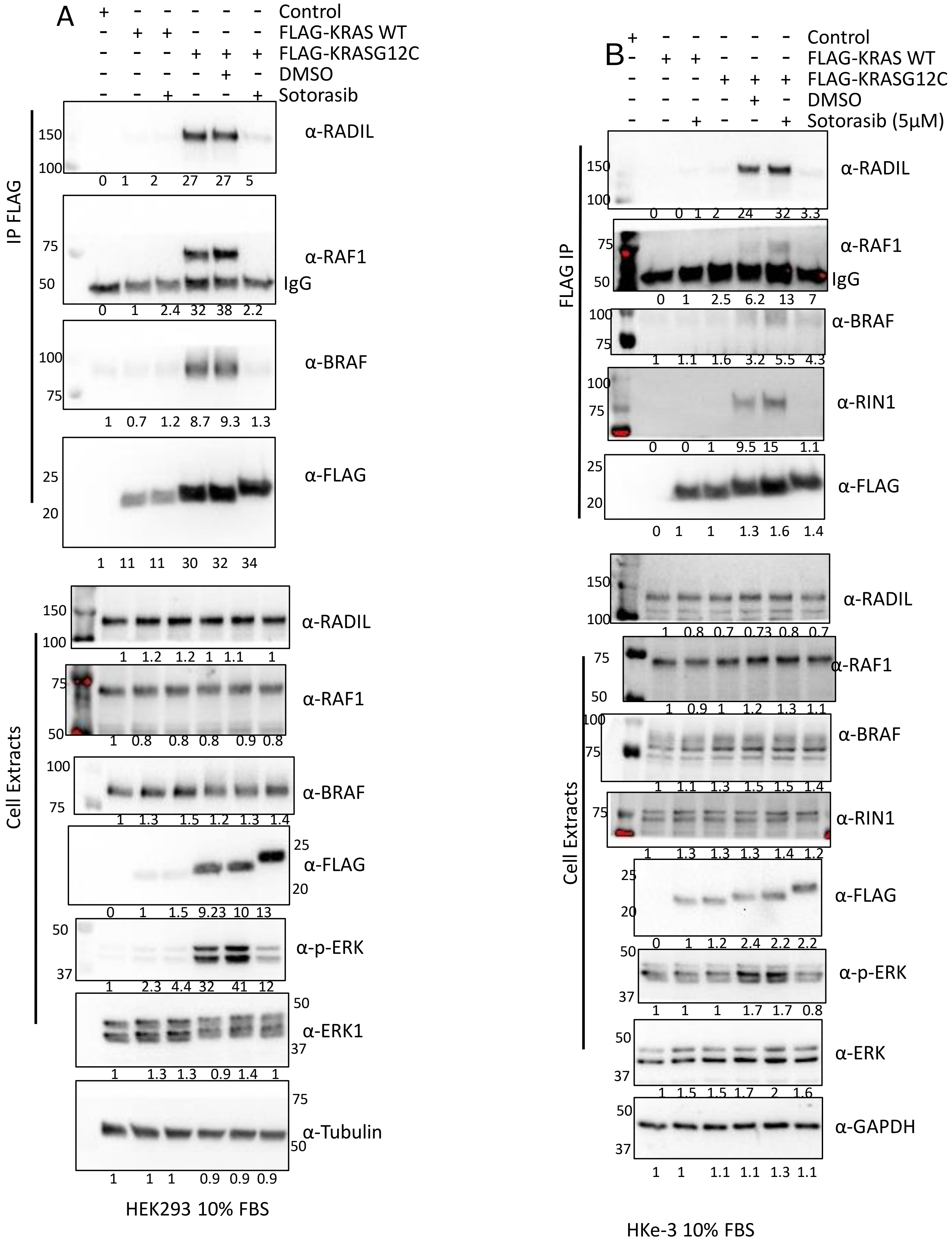

Original Blots and quantification for Figure 8 cont

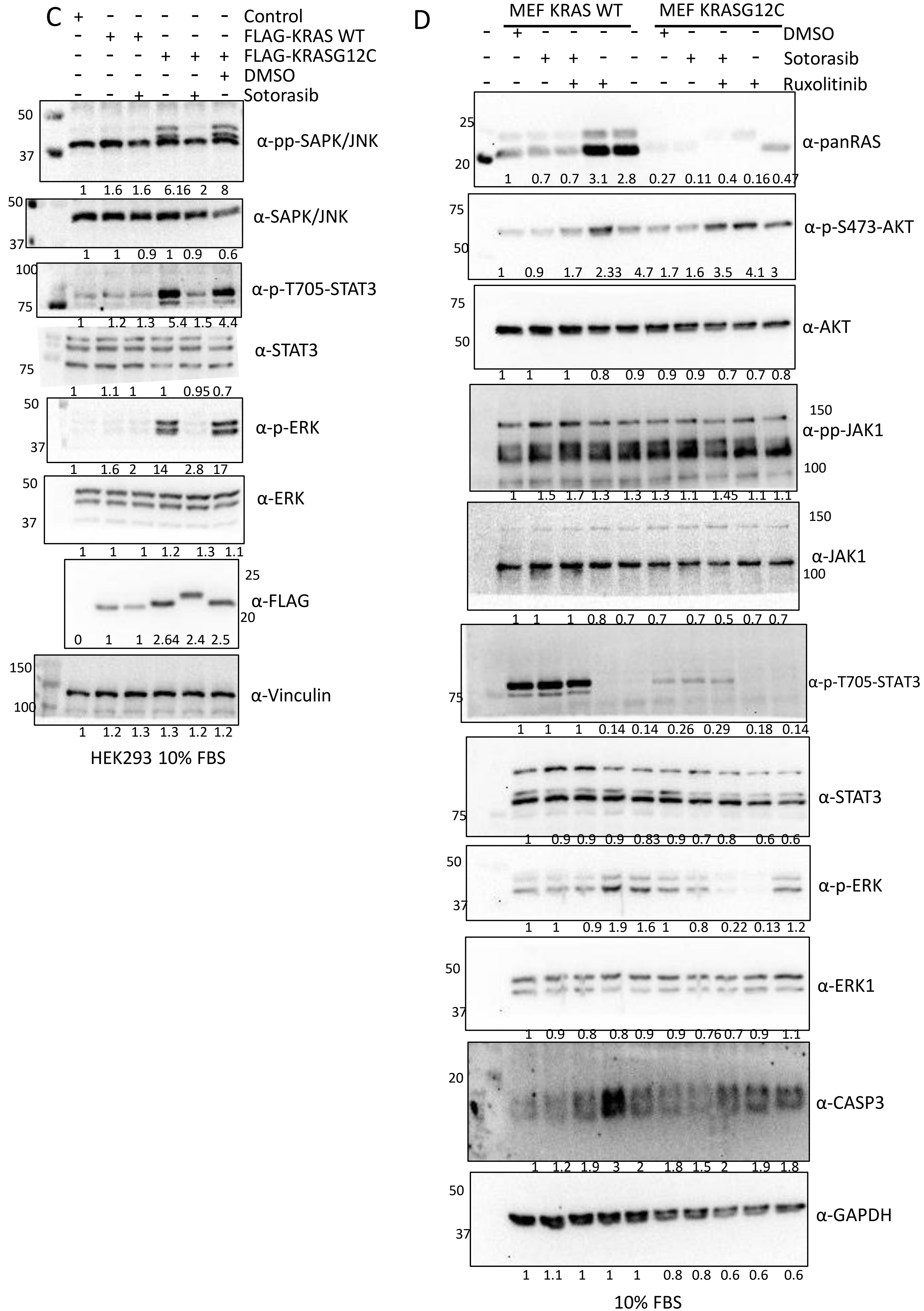

Original Blots and quantification for Figure S5

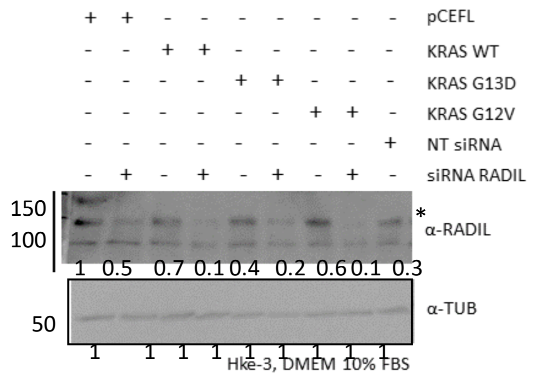

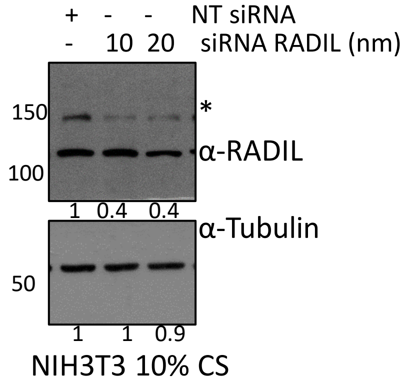

Original Blots and quantification for Figure S6

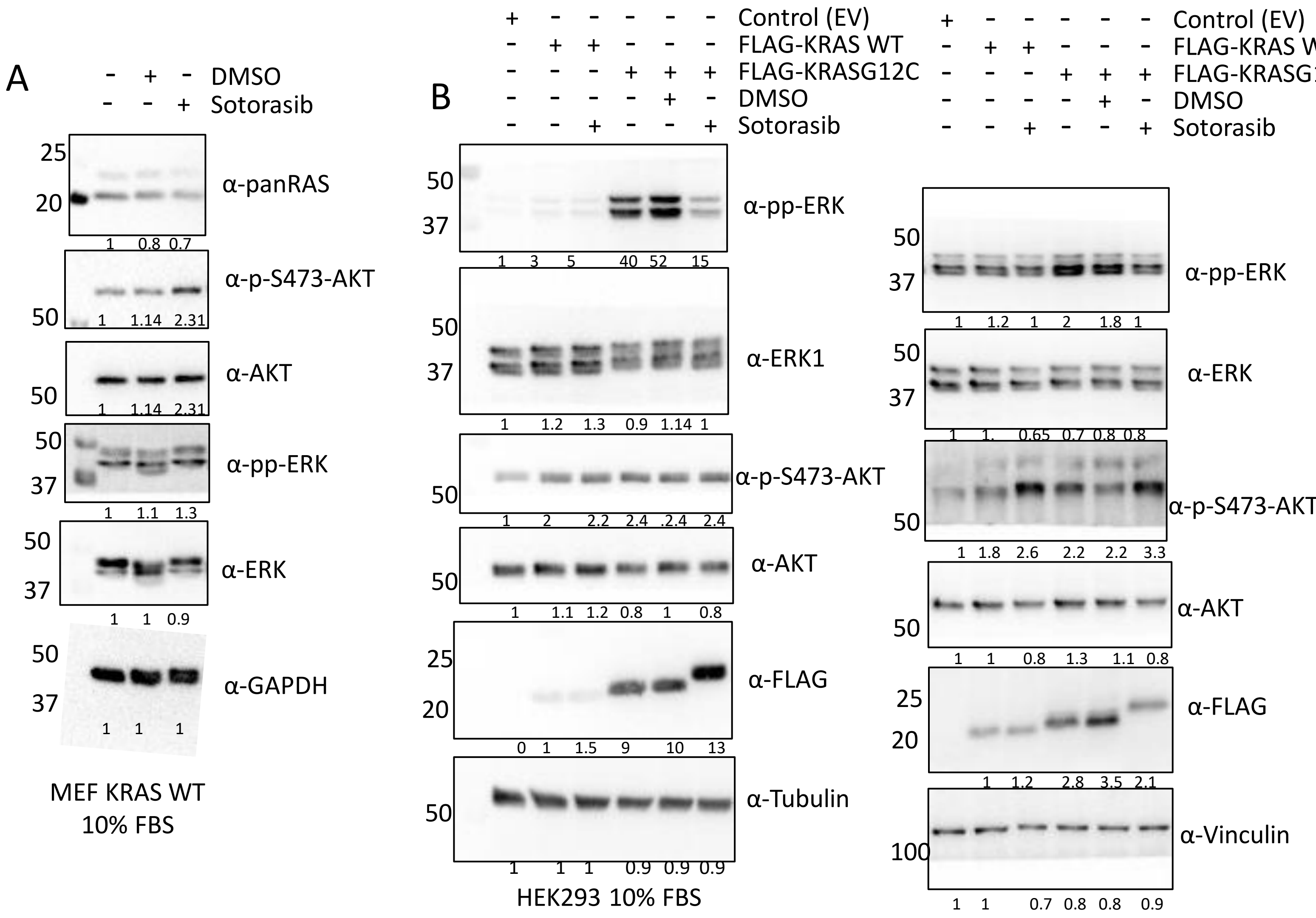

Original Blots and quantification for Figure S7

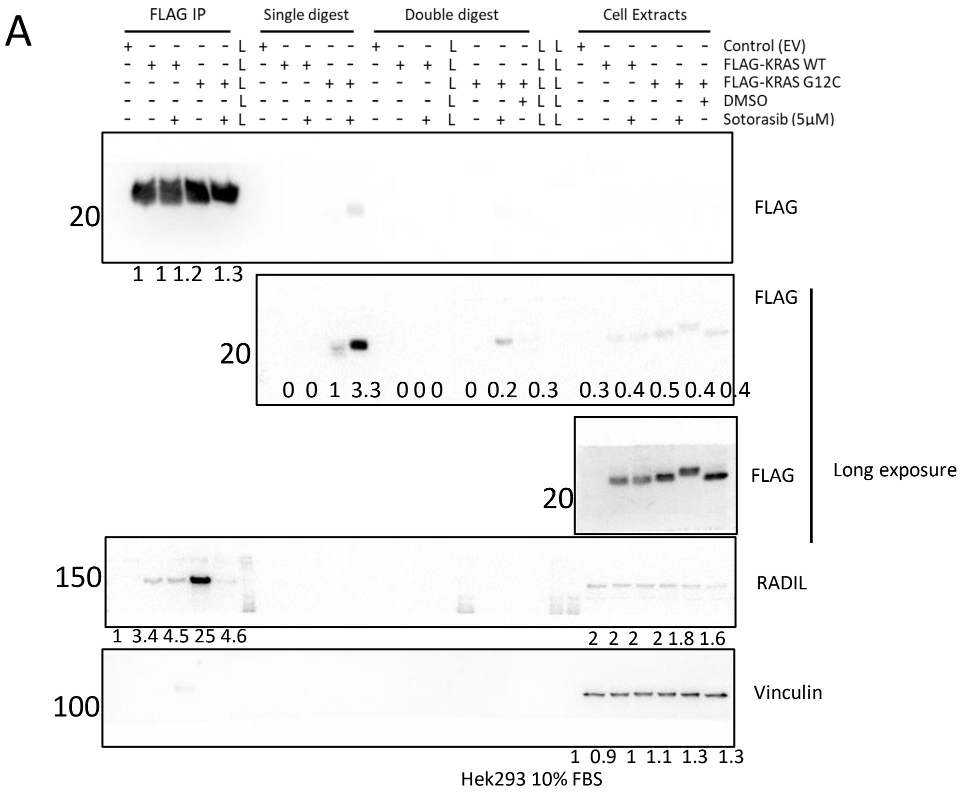

Supplement: Supplementary file 1 [file cancers-15-04141-s001.zip › supplementary figures.pdf]
